# Analysis of the foam-forming of non-woven lightweight fibrous materials using X-ray tomography

**DOI:** 10.1007/s42452-021-04172-9

**Published:** 2021-01-24

**Authors:** S. R. Burke, M. E. Möbius, T. Hjelt, J. A. Ketoja, S. Hutzler

**Affiliations:** 1 School of Physics, Trinity College Dublin, The University of Dublin, Dublin, Ireland; 2 VTT Technical Research Centre of Finland Ltd., Espoo, Finland

**Keywords:** Lightweight fibrous materials, Foam-forming, Natural fibres, Fibre orientation, Compressive strength, Imaging, Materials

## Abstract

**Abstract:**

Foam-forming has in the past predominantly been used to create two-dimensional sheet-like fibrous materials. Allowing the foam to drain freely and decay under gravity, rather than applying a vacuum to remove it rapidly, we can produce lightweight three-dimensional fibrous structures from cellulose fibres, of potential use for thermal and acoustic insulation. $\mu$CT scanning of the fibrous materials enable us to determine both void size distributions and also distributions of fibre orientations. Through image analysis and uniaxial compression testing, we find that the orientation of the fibres, rather than the size of the voids, determine the compressive strength of the material. The fibrous samples display a layering of the fibres perpendicular to the direction of drainage of the precursor liquid foam. This leads to an anisotropy of the compressive behaviour of the samples. Varying the initial liquid fraction of the foam allows for tuning of the compressive strength. We show an increase in over seven times can be achieved for samples of the same density (13 kg.m^-3^).

**Graphic abstract:**

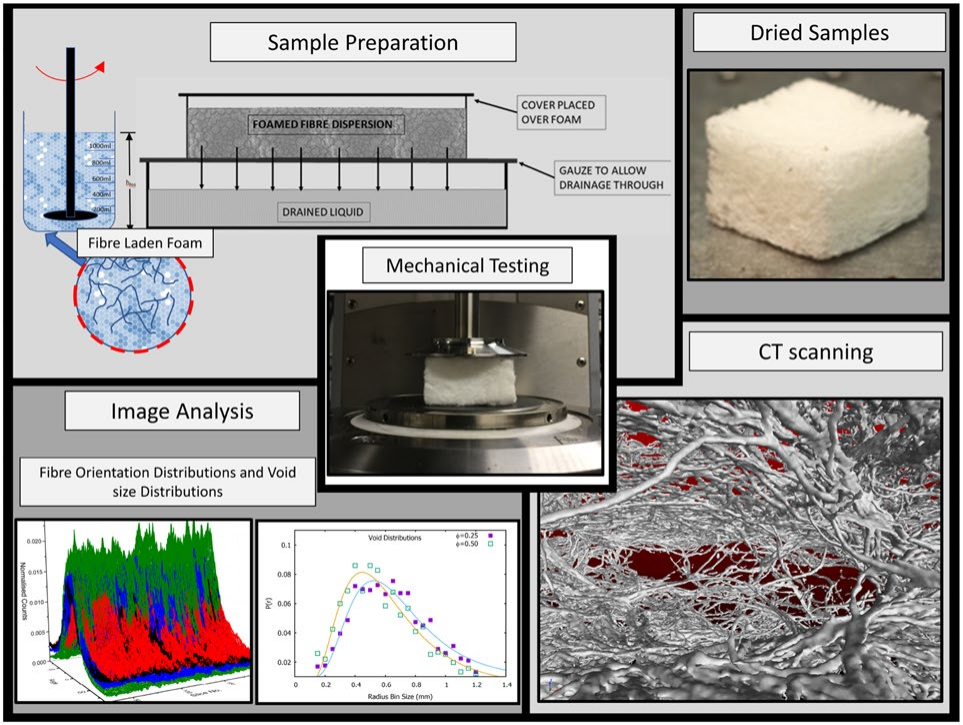

## Introduction

There is an increased drive to replace lightweight products derived from petrochemicals, such as expanded polystyrene, polyurethane and phenolic foam with new, sustainable and environmentally friendly materials. Low density materials made using natural fibres can play a role in this endeavour, provided it is possible to match the properties of the existing materials in relation to thermal [1] and acoustic [2] insulation, fire retardancy and mechanical strength for packing materials [3, 4]. The foam-forming technique may offer a promising route to create non-woven natural materials from cellulose, peat or spent grain, with characteristics that can be tuned via controlling properties of the foam used for their production.

First developed in the 1960’s [5], the foam-forming technique uses foam as a dispersal medium for fibres. Fibres are initially dispersed in water by mechanical shearing. A surfactant is then added and the dispersion is sheared once again, entraining air bubbles into the dispersion, thus resulting in a liquid foam containing fibres. The fibre-foam dispersion is then poured into a drainage vessel with a wire gauze as a base. The foam is typically collapsed by applying a vacuum to the underside of the gauze, leaving a thin evenly-dispersed sheet of fibres on the gauze [6].

**Fig. 1:**
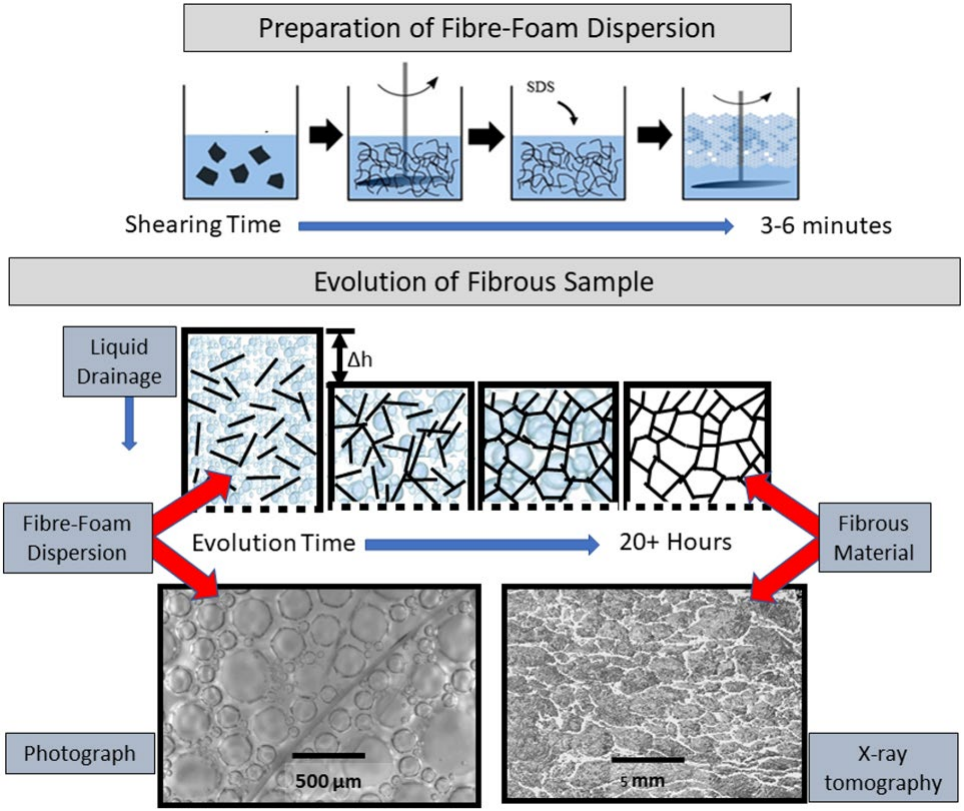
Schematics illustrating how a fibre-foam dispersion is prepared and then slowly evolves into a dry expanded, non-woven fibrous material. (For details see text)

Here we describe a variation of this technique which enables the creation of lightweight bulky structures. Rather than applying a vacuum, the liquid contained in the foam is allowed to drain freely from the dispersion through the gauze due to gravity. As the foam slowly decays as a consequence of drainage and evaporation, it leaves behind a low density bulky structure in the form of a network of fibres. Figure 1 shows a schematic of the formation process.

Unlike in the recent work by [7] neither the wet foam fibre dispersions nor the dried sample are compressed. This results in samples of density 13 kg.m^-3^ compared with densities of about 40 kg.m^-3^ by [7]. While Pöhler et al. use X-ray tomography mainly to illustrate inhomogeneities in the sample we use the technique to characterise and quantify the internal structure of our fibre networks.

Details of the sample preparation are given in Sect. 2, together with a description of the CT scanning technique that we used to image our samples. In Sect. 3 we characterize the fibrous materials in terms of density distributions and fibre orientation. The average void size in the samples is related to the average bubble size in the precursor foam-fibre dispersions. In Sect. 4 we report compression testing on our samples. An interpretation of the found anisotropy due to fibre orientation is given in Sect. 5. Section 6 is a brief summary of our main results.

X-ray tomography confirms the role that foam drainage has for fibre alignment and how this in turn determines mechanical stiffness of the dried fibrous material. This, together with the identification of the average void size, is important for the tailoring of these materials for particular applications.

## Sample preparation and imaging

### Sample preparation

All samples were made with dried Northern Bleached Softwood Kraft fibres (Stora Enso, Imatra, Finland) of average length 2.0±0.1mm and diameter of $35\pm 5\, \mu \hbox {m}$ [8]. The fibres were soaked in water for 24 hours before shearing them with a mixing disk to create an aqueous dispersion [9]. Fibre content was kept at a constant 7g in all dispersions. Surfactant is added to the aqueous dispersion which is then sheared once again to produce the fibre-foam dispersion, which we then pour into a drainage vessel. The final volume of all fibre-foam dispersions was 1200ml. We varied the initial liquid volume fraction, $\phi _{i}$, of the foam by varying the amount of water that the fibres were dispersed in (300 to 600ml, giving a range of $\phi _{i}$ from 0.25 to 0.50). We will see below that $\phi _{i}$ is an important process parameter which controls fibre orientation and thus mechanical strength of the dry fibrous material.

Our choice of surfactant was sodium dodecyl sulfate SDS, (98% purity supplied by Sigma Aldrich), at a concentration of 6.5g/L, therefore above the critical micelle concentration of 2.3 g/L. The drainage vessel has a fine wire gauze as a base to allow the liquid to drain through (mesh size $50 \, \mu \rm m$). After about 48 hours at room temperature the foam has entirely disappeared due to evaporation-driven rupture of its liquid films and the emerging fibre network is dry. It is then removed from the container and cut into sample sizes (33 x 33 x 16 mm).

### CT scanning of foam-formed fibrous materials

In order to image our dry fibrous samples and subsequently perform a fibre orientation analysis, CT scanning was used to probe the interior structure non-invasively.

X-ray image acquisition was performed with a Nikon XTH 225 ST device, fitted with a reflection target. Our initial scans revealed that our samples had low X-ray attenuation due to the high porosity/ low density of the material. To obtain high contrast images we used Molybdenum as a target material, along with a low electron beam energy (45 kV) and a beam current of 200 mA. Molybdenum produces low energy X-rays resulting in greater beam attenuation. Appendix A describes details of the scanning procedure.

**Fig. 2:**
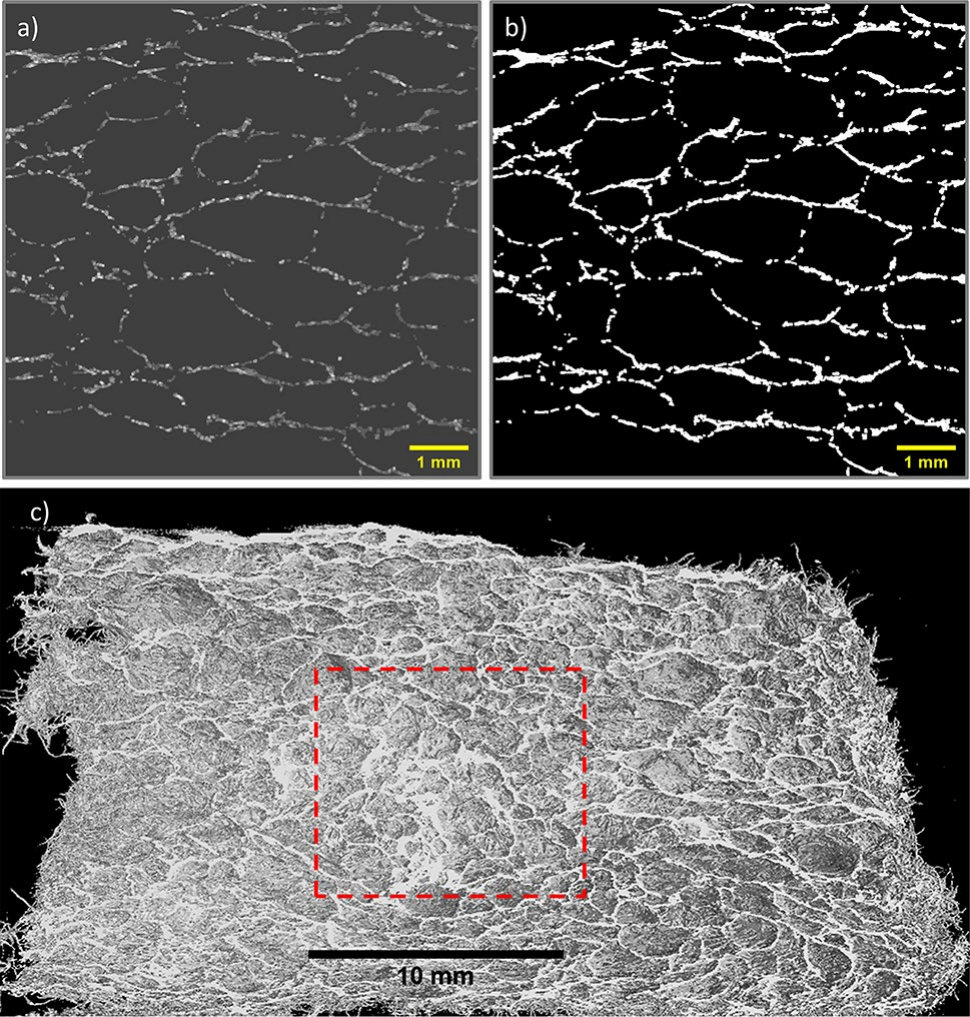
**a** A raw greyscale image slice from the x-direction. **b** Isodata thresholding applied to a raw image slice. **c** A volume rendering of one of the lightweight foam-formed fibrous materials. The red-dashed square represents the cube size (edge length = 10 mm) our analysis was performed on. The front half of the structure has been removed using software manipulation, revealing the internal fibre network and void spacing of the material

Figure 2 shows a three-dimensional rendered image of one of the samples where the front half has been removed via software manipulation. The clear visibility of the individual fibres enables us to both analyse sample porosity (sect. 3.2) and fibre orientation (sect. 3.3).

## Sample characterisation

**Fig. 3:**
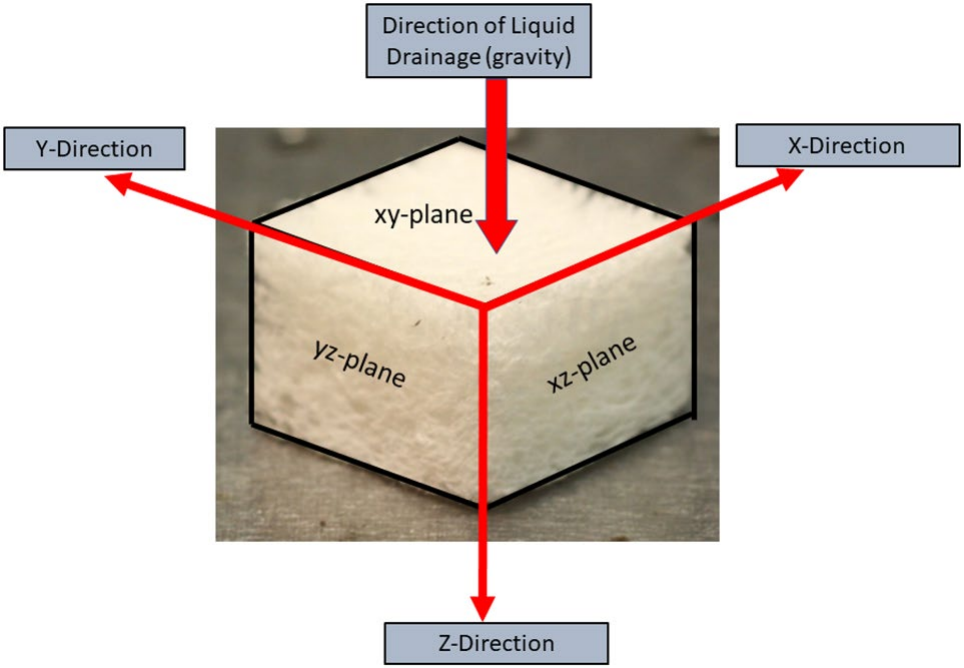
Gravity acting in the z-direction plays a role in foam drainage, partial sample collapse and fibre orientation. Compression tests (Sect. 4) were carried out in x, y, and z-directions

In the following sections we refer to the z-direction as the direction in which gravity acts during sample formation. This is the net flow direction of liquid as the fibre-foam dispersion dries out and it is also the direction of (partial) weight collapse of the sample. Figure 3 shows the corresponding coordinate system.

### Density profiles

**Fig. 4:**
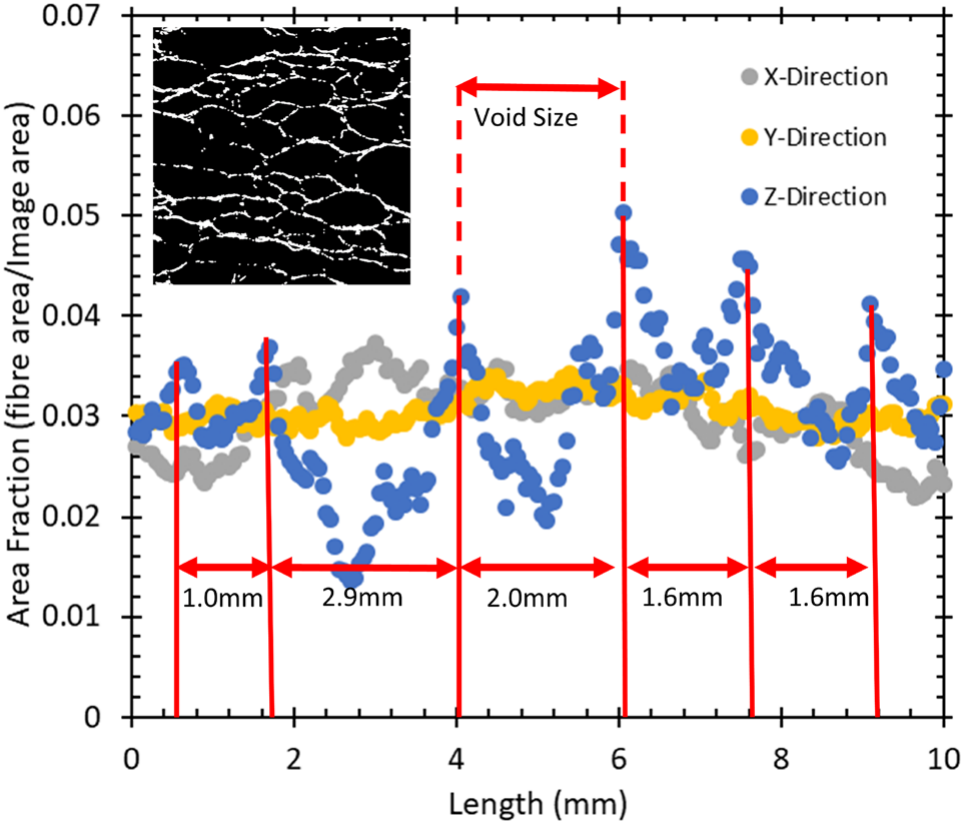
Density profiles of one of our foam-formed fibrous materials. Plotted is the fibre area fraction as a function of distance through the sample in mm. The x and y-directions display an approximately uniform density profile throughout the sample. The variations in the z-direction indicates layering of the fibres (see Sect. 4). The majority of the fibres is in the xy-plane (horizontal) with the remainder of the fibres supporting these layers. The inset shows a binarized image of a vertical sample slice

Our first analysis of the orthogonal image stacks concerned the computation of the density profiles of the structure from along each axial direction, e.g. the z-direction refers to the images that lie in the xy-plane. We analysed 200 regularly spaced images (spacing 0.05mm) from each image stack. Images were cropped to size (10 x 10 mm) and then binarized with the isodata method and global thresholding applied (see inset of Fig. 4), after which the fibre area fractions were obtained for each image. In Fig. 9 (Sect. 3.3) the white pixels represent the fibres and the black represent the void space. The fibre area fraction, $A_{f}$, is defined as the ratio of the number of white pixels to the total pixel number.

Figure 4 shows fibre area fractions along a sample in the three orthogonal directions x,y,z. While the profiles along the x and y-directions appear roughly uniform the z-direction reveals a number of sharp peaks and troughs. These are indicative of the layering of fibres with a spacing of about 1.8 ± 0.2 mm, which we will discuss in Sect. 3.3.

### Relation between bubble size in the dispersion and the void size of the fibrous sample

**Fig. 5:**
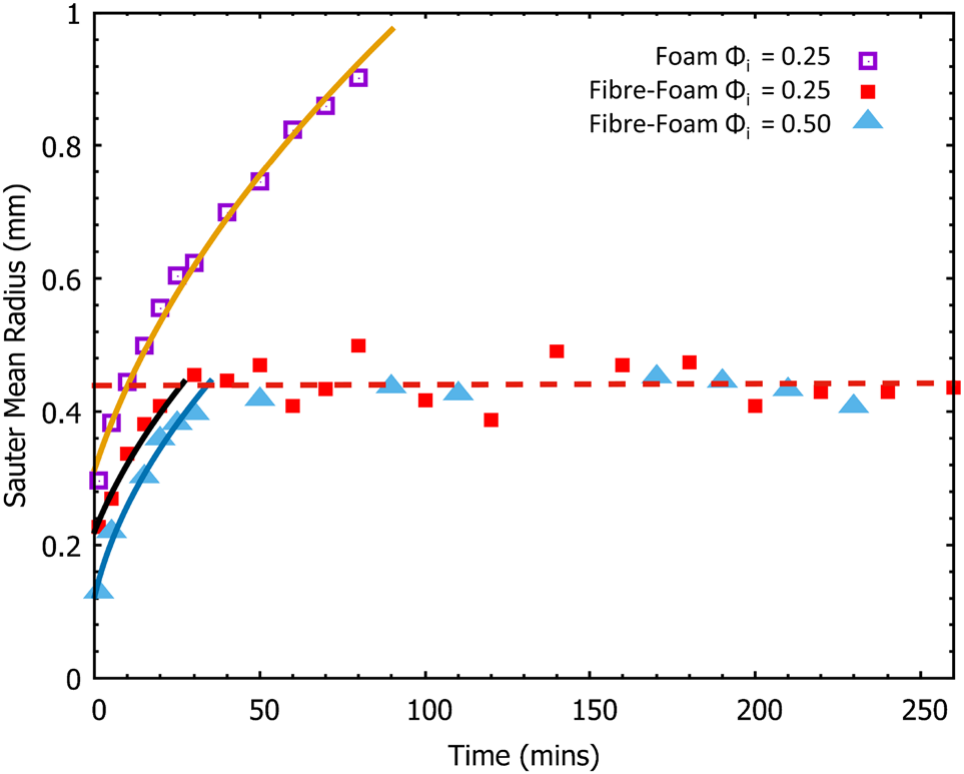
Sauter Mean Radius for a pure foam and two fibre-foam dispersions as a function of time. The pure foam had an initial liquid fraction of 0.25. Both fibre-foam dispersions contained the same mass of fibres with initial liquid fractions 0.25 and 0.50 respectively. The pure foam continues to coarsen until there is no foam remaining, whereas where fibres have been added, coarsening ceases after approximately 30 minutes, regardless of the initial liquid fraction. Solid lines are fits to the coarsening law, $r_{32}\propto (t-t_{o})^{1/2}$

The initial average bubble radius in our freshly produced fibre-foam dispersions is about 200$\, \mu$m (see Fig. 5), while the layering of fibres exceeds 1mm. A pure foam is known to coarsen as the higher-pressure gas in smaller bubbles diffuses into the larger neighbouring bubbles through the thin liquid film that separates them. As a result of coarsening the number of bubbles in the foam continually decreases, while the average bubble size increases with time *t*, proportional to $(t-t_{o})^{1/2}$, where $t_o$ is an offset [10]. The addition of fibres leads to a slow-down in coarsening of a foam [11].

We note that also the initial bubble size is reduced; for a fixed rotation speed of the mixing disk the addition of fibres results in extra shear forces exerted by the fibres during mixing [12], leading to smaller bubbles.

Here we provide a detailed study of foam coarsening and also relate bubble size distributions to void size distributions in the foam-formed fibrous materials. We have measured the bubble size distributions for the full lifetime of a pure foam (up to 90 minutes) and up to 1400 minutes for the fibre-foam dispersions.

#### Bubble size in a coarsening fibre-foam dispersion

In our first experiment we compared coarsening in a pure foam (initial liquid fraction $\phi _{i}=0.25$) and two fibre-foam dispersions (liquid fractions of $\phi _{i}=0.25$ and $\phi _{i}=0.50$ respectively), each containing 7g of Kraft fibres. The foams were poured into containers that had a gauze as a base, allowing the liquid to drain away. Samples of the foams were taken at regular intervals (5 minutes intervals during the initial rapid growth regime, increasing to every 20 minutes at later times, see Fig. 5) by inserting two microscope slides (separated with a spacing of 0.2mm) into the dispersions and trapping a sample of foam between them. An image of the slide was taken and the area of each bubble was determined using imageJ. The volume of each bubble was calculated from the area measurements and the known slide spacing, allowing us to infer an equivalent sphere radius for each bubble [11, 13]. The number of bubbles measured varied initially from  7000 per image to about  1000 in the coarsened foam sample. As bubble growth increased less bubbles were included in the analysis.

Foam coarsening is best expressed in the variation of the Sauter mean radius $r_{32}$ as a function of time [14]. It is defined as $r_{32} = \left\langle r^3 \right\rangle / \left\langle r^2 \right\rangle$. The Sauter mean radius thus emphasizes the importance of bubble surface area in inter-bubble gas diffusion.

Figure 5 shows that $r_{32}$ of the pure foam continually increases throughout the foam lifetime, up until 90 minutes, when all foam has decayed. In contrast, when fibres are added to a foam, the initial bubble growth comes to a halt after about 30 minutes, resulting in a Sauter mean radius of about 0.4 $\pm \: 0.1\, \hbox {mm}$ for both values of initial liquid fraction. The fibres are acting as pinning sites for the bubbles films, limiting further growth [11]. The growth regime for all three data sets is described by $r(t)\propto (t-t_{0})^{0.5}$.

The lifetime of the fibre-foam dispersion is far in excess of the 260 minutes displayed in Fig. 5, which still contained bubbles after 23 hours. This extended lifetime may in part be due to the fibres increasing the relative humidity within the dispersion by trapping the vapour layer that forms over liquid during evaporation. Bubble films thin due to liquid drainage and evaporation and eventually rupture. Therefore by keeping the films in a more humid environment the rate of evaporation may be reduced, thus extending their lifetime.

#### Comparison of bubble and void sizes

How does the average long time bubble radius of 0.4mm in the fibre-foam dispersion compare with the average void size in the foam-formed dried material? In order to investigate this we carried out a void size analysis using X-ray data of two fibrous samples (density 13 kg.m^-3^) created from fibre-foam dispersions with initial liquid fractions of $\phi _{i}$=0.25 and $\phi _{i}$=0.50. We compute a void size distribution from two-dimensional cross-sections of the sample. In general the result is different from the actual three-dimensional void size distribution. However, the 2D analysis is sufficient to compare different samples. Moreover, the average void size will be of the same order of magnitude in both 2D and 3D.

**Fig. 6:**
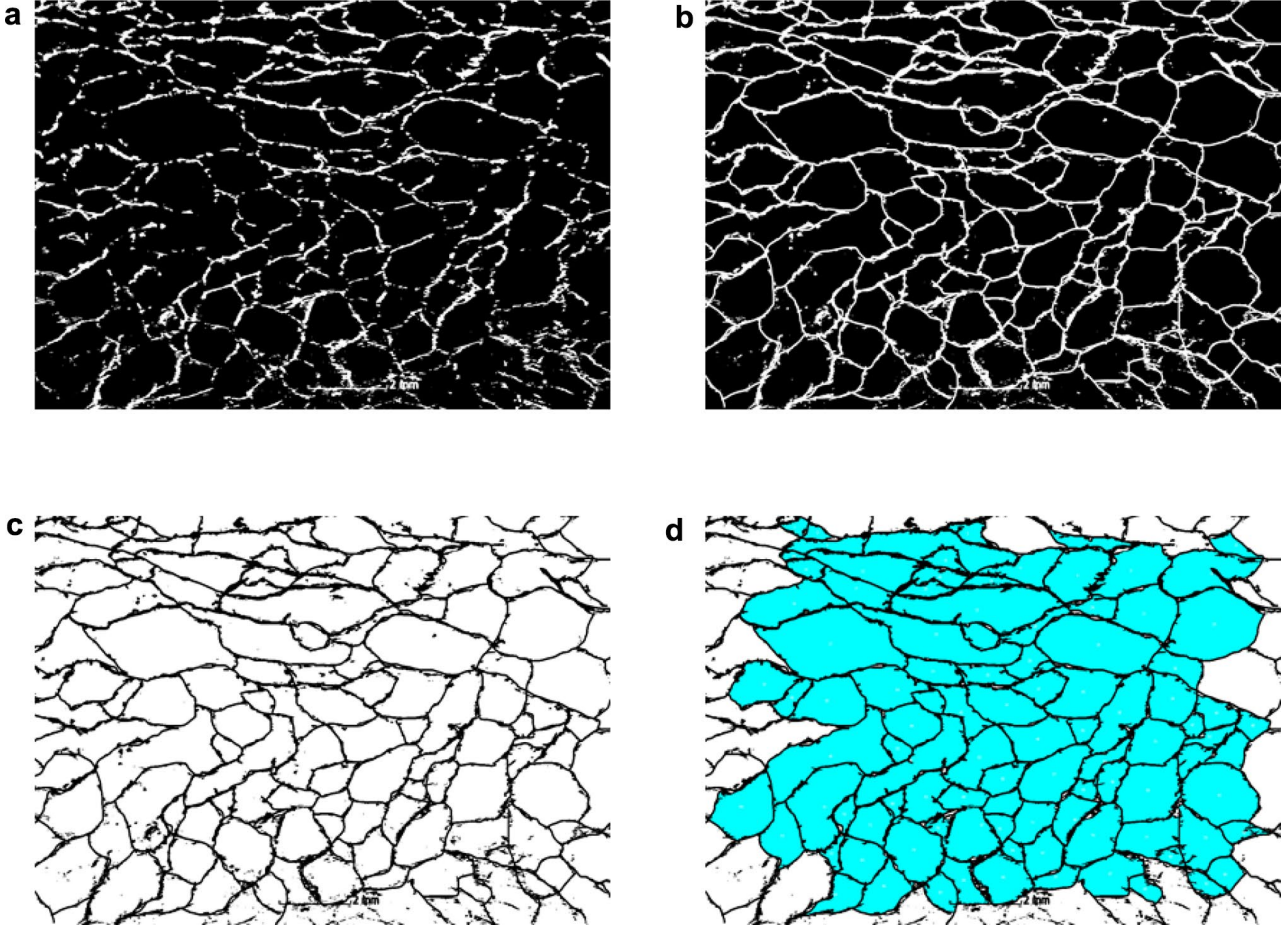
One of the images viewed from the x-direction that is used for a void size analysis. **a** A single binarized image slice of one sample **b** Voids are manually closed by connecting gaps between fibres in close contact with each other. **c** Inverted image prior to performing area analysis. **d** Area analysis output from imageJ particle analysis function. Void area along image edges are excluded from the analysis

**Fig. 7:**
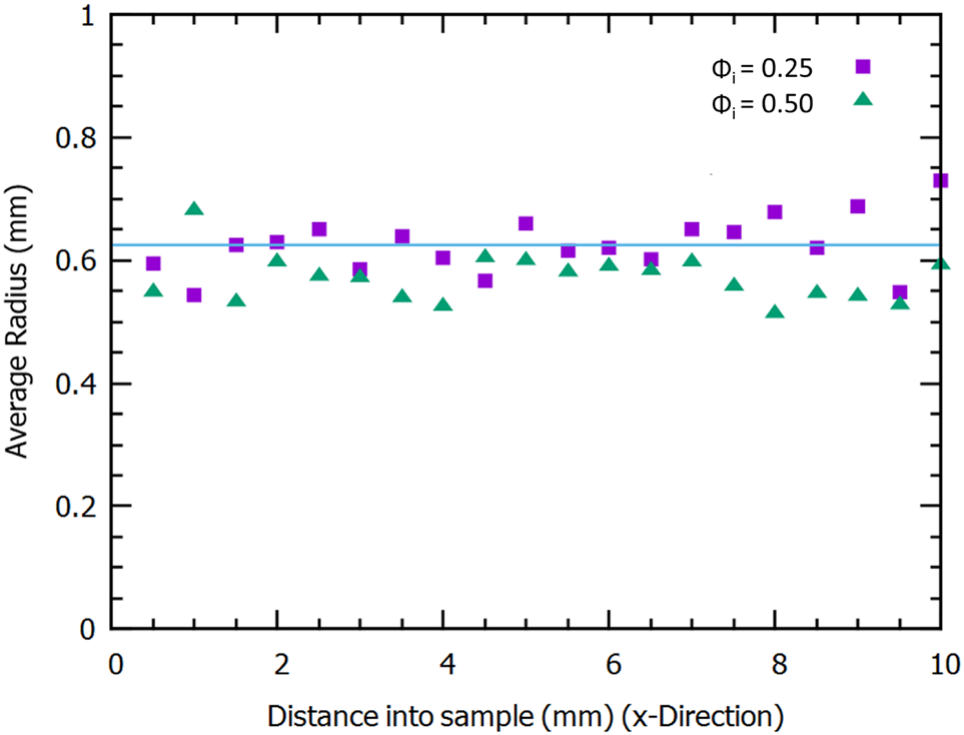
Average void radii, measured along the x-direction of two foam-formed samples produced for $\phi _{i}$ = 0.25 and 0.50. The average void size is approximately 1.5 times as large as the average bubble size, $r_{32}\approx 0.4\, \rm mm$ (see Fig. 5)

The analysis was performed on twenty images, taken from the x-direction, for each sample (image spacing: 0.5 mm). Images were first binarized (isodata method from ImageJ with global thresholding applied) an then the voids were manually closed by closing gaps in the fibres surrounding the void with their nearest neighbours. A particle area size analysis was performed on each image using imageJ’s particle analysis function. The total number of voids analysed was almost 2000 per sample. We express the size of a void in terms of the radius of a circle with the same area as the cross-section of the void. Figure 6 shows the workflow for one image. Figure 7 shows that the average void size, measured along the x-direction, is roughly constant, its value of $r_{v}$=0.6±0.2mm is also independent of the value of initial liquid fraction. Average void size thus exceeds the average bubble size by approximately 50$\%$, as determined in the regime where foam coarsening is arrested by the presence of fibres. Bubble coalescence during the drying process may explain the larger void size.

**Fig. 8:**
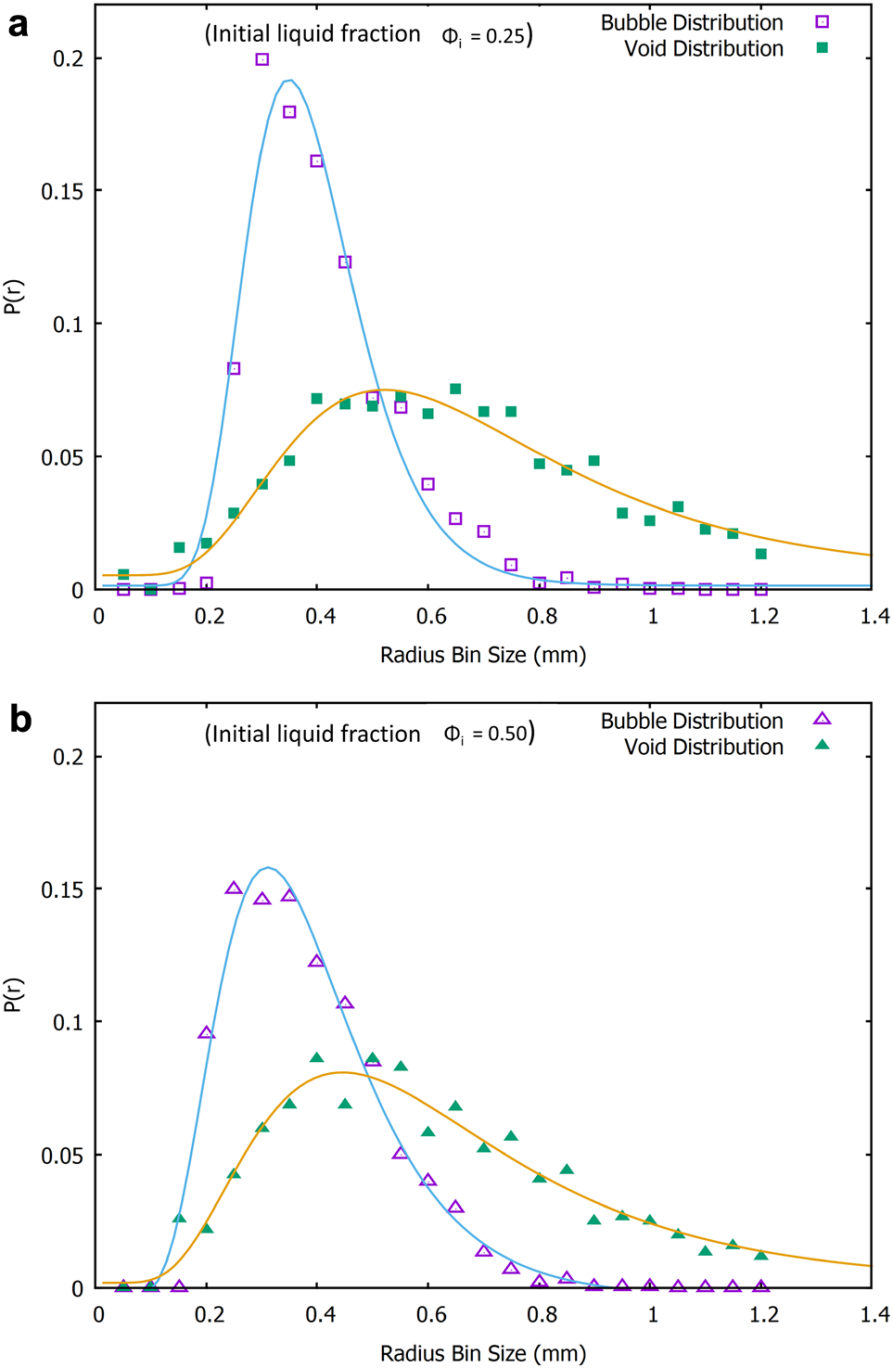
Bubble and void radii distributions for the samples made with initial liquid fraction a) $\phi _{i} = 0.25$, b) $\phi _{i} = 0.50$. Solid lines are fits to log-normal distributions

We have also compiled the respective distributions of both void and bubble size, see Fig. 8. Both are well described by log-normal distributions.

Average bubble size in the coarsening-arrested fibre-foam dispersion has thus emerged as controlling the average void size of the fibrous networks. Burke et al. [9] showed that the initial liquid fraction plays a role in determining the compressive modulus of the networks. In the following we will demonstrate, again using our X-ray data, that this is due to its effect on fibre *orientation*.

### Determination and interpretation of fibre orientation

Fibre orientation analysis was carried out on four samples, each of density around 13 kg.m^-3^, but produced from fibre-foam dispersions with four different values for initial liquid fraction $\phi _{i}$ (0.25, 0.33, 0.42, 0.50). Increasing $\phi _{i}$ leads to an increased volume of liquid draining through the dispersion in the initial stage of sample preparation, with the effect of aligning more of the fibres into the direction of drainage (z-direction). In Sect. 4 we will show how this affects the compressive strength of the structures.

**Fig. 9:**
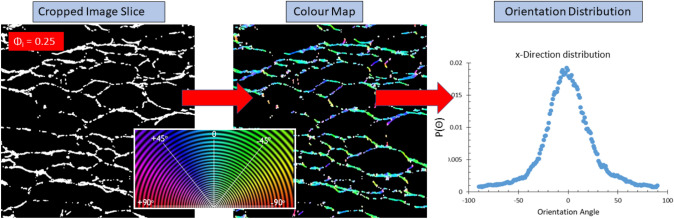
Schematic of OrientationJ’s workflow for a cropped image slice obtained from a sample produced with $\phi _{i}=0.25$. The algorithm returns a colour map, as well as an orientation distribution for each image. The colour wheel in the centre shows the colours assigned to each orientation angle in the colour maps, the $0^{\circ }$ angle being perpendicular to the direction of gravity and drainage

The fibre orientation analysis was performed on the greyscale image stacks of the samples (obtained by CT scanning) with the ImageJ/Fiji plugin OrientationJ [15]. Each image stack contained 200 cropped images (10 x 10 mm see Fig. 2), separated by a spacing of 0.05mm. This equates to a total distance through the sample of 10mm. OrientationJ is specifically designed to analyse the isotropic and orientational properties of a 2D image. The plugin computes a histogram of orientation distributions for each image (in our case 200 images per direction per sample), see Fig. 9 for a schematic of the workflow, and Appendix B for details of the algorithm. The resultant orientation distribution $P(\theta )$ is a 2D projection of the fibre orientations.

**Fig. 10:**
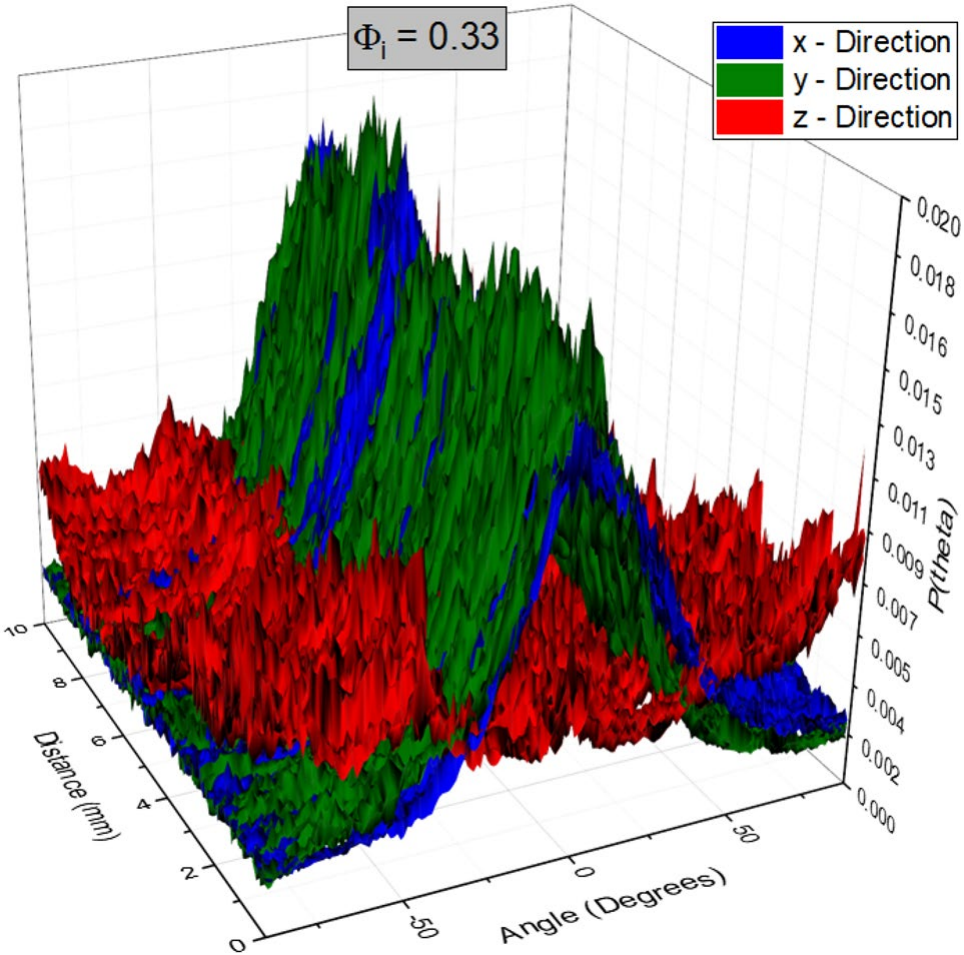
Fibre orientation distributions for the sample made from a fibre-foam dispersion with $\phi _{i} = 0.33$. The angle in degrees, ranging from $-90^{\circ }$ to $+90^{\circ }$ is shown on the x-axis, distance (depth within the sample) is shown on the y-axis. The normalised counts are represented on the z-axis. The distributions from the x and y directions (blue and green data sets) are seen to have one dominant peak through the sample located at angle zero, indicating the layering of fibres along the horizontal perpendicular to gravity. No dominant direction is revealed when probing from the z-direction (red data set)

Figure 10 shows three fibre orientation distributions (along the x, y and z-directions) for the sample produced with $\phi _{i}=0.33$. The distributions along both the x and y-directions feature a distinct peak at angle zero throughout the sample, corresponding to a fibre orientation perpendicular to the direction of gravity. This corresponds to the majority of fibres lying in the xy-plane. The red data set represents the distributions along the z-direction (xy-plane). Unlike the x and y directions, no single dominant direction was observed.

**Fig. 11:**
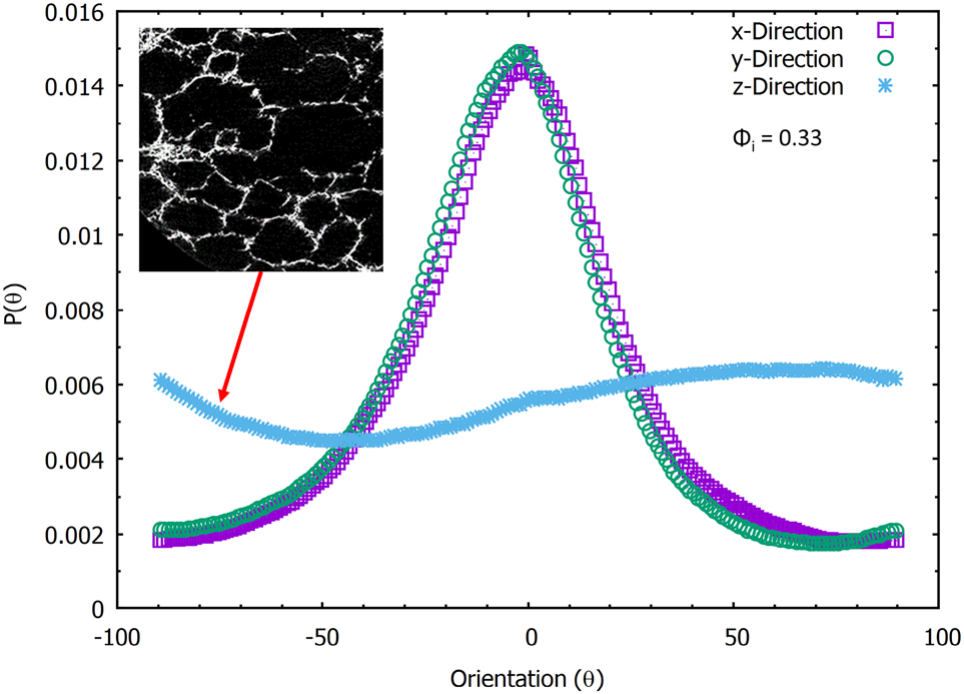
The averaged fibre orientations for the 3 directions. The views from both x and y directions display a single dominant angle at zero degrees, and very similar widths, indicating a preferred alignment in the horizontal plane perpendicular to drainage. The distribution along the z-direction is nearly flat. Any small variation with angle is due to finite size sampling

We have also averaged the distributions of all the image slices of Fig. 10 along the 3 directions as shown in Fig. 11. The distributions viewed from both x and y directions are similar due to symmetry and feature a peak at angle zero corresponding to horizontal orientation of the fibres; viewing the sample from the z-direction results in nearly flat distribution indicating no preferred fibre alignment in the horizontal plane.

**Fig. 12:**
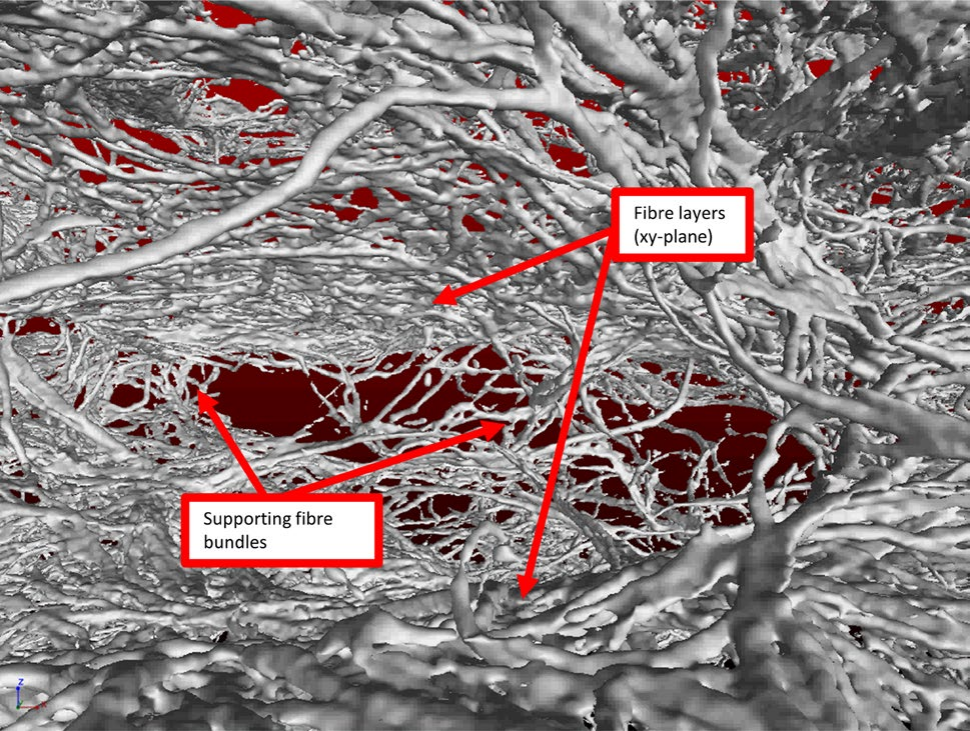
Reconstructed view from inside one of the voids within a sample. The image shows two fibre layers, orientated in the xy-plane being supported and kept apart by fibre bundles. When compressing the sample along the z-direction it is these fibre bundles which distribute the load onto the layers (see Sect. 4). The fibres have an average length of $2.0\pm 0.1\hbox {mm}$ and diameter of $35\mu \hbox {m}$

**Fig. 13:**
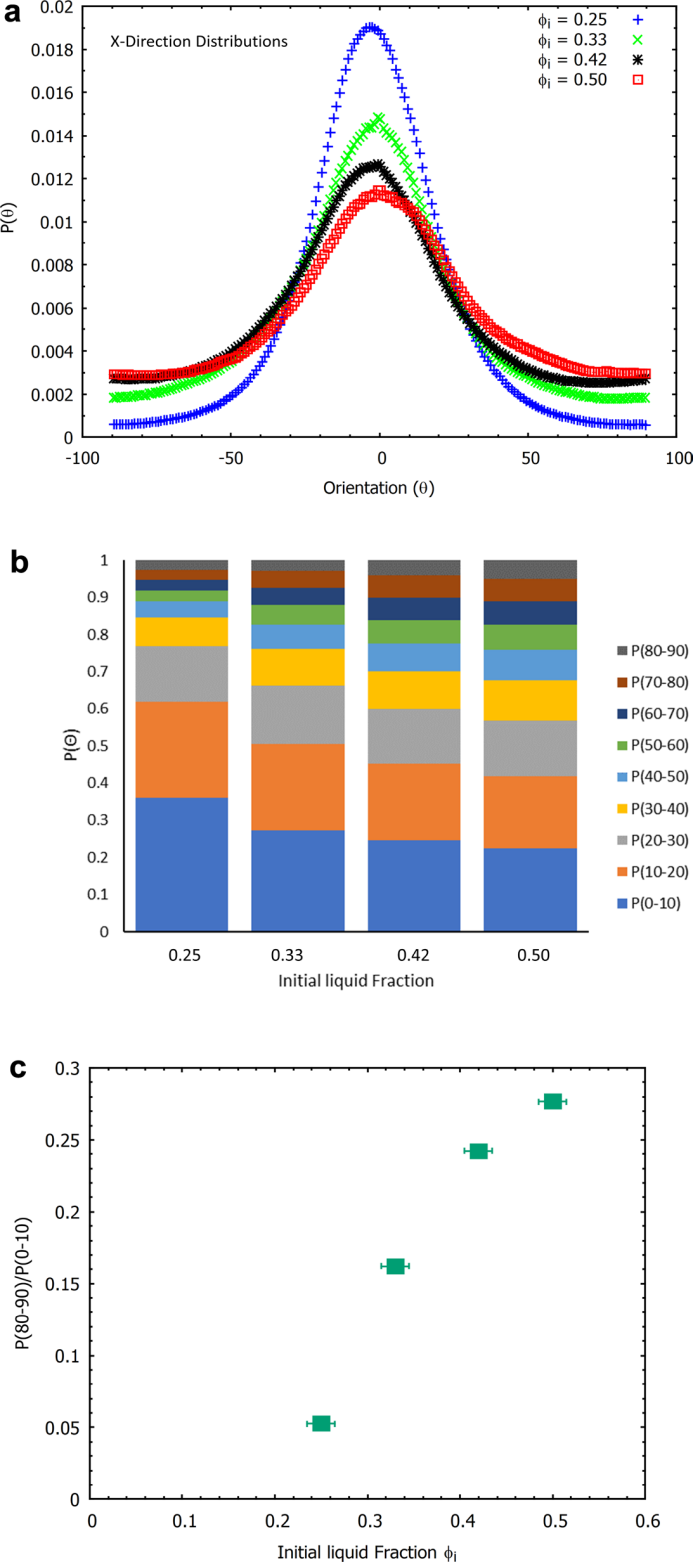
**a** A two-dimensional projection of the distributions along the x-direction for samples produced using four different values of initial liquid fraction $\phi _{i}$. The width of the distributions increases with $\phi _{i}$. **b** Fibre orientation probabilities for a range of bin sizes as a function of the initial liquid fraction. Fraction of fibres orientated with angles 0$^{\circ }$ to 10$^{\circ }$, 10$^{\circ }$ to 20$^{\circ }$, 20$^{\circ }$ to 30$^{\circ }$ etc. Increasing the initial liquid fraction $\phi _{i}$ leads to more fibres aligning out of the xy-plane.** c** Probability ratio of $P(80-90)/P(0-10)$ as a function of $\phi _{i}$. An increase in initial liquid fraction results in more fibres aligning in the direction of gravity

The layering of the fibres in the xy-plane can be observed in a full 3D rendering of the X-ray data, see Fig. 12 and movie (supplemental material). It also indicates a flattening of the voids, following the partial sample collapse. The distributions for the z-direction show a much broader distribution, due to the fibres being orientated around the bubbles while in the foam. Since the orientation distributions, as obtained from scanning the sample in both x and y-directions, are very similar (as expected by symmetry) we will in the following only consider distributions from the x-direction. Figure 13 shows the averaged distributions $P(\theta )$ for all our four samples, produced from different values of initial liquid fraction, but having the same material density.

While the maxima of the four distributions are all at angle zero (i.e. a preferred fibre orientation in the x-y plane) the distributions broaden with increasing initial liquid fraction, i.e. fibres align increasingly out of the horizontal x-y plane.

Figure 13 a shows P($\theta$) as a function of initial liquid fraction $\phi _{i}$. The symmetry of the distributions allows us to combine the data for positive and negative angles. The bins in Fig. 13b were computed by integrating over the corresponding ranges of the angles. The $P(0-10)$ bin size shows while the majority of fibres lie in this range, the fraction decreases with an increase in $\phi _{i}$. Similar trends are seen for the $P(10-20)$ and $P(20-30)$ ranges, after which the trend is reversed, i.e. we have an increasing probability of fibres being orientated towards the z-direction (direction of gravity). This is illustrated in Fig. 13c, which shows the ratio $P(80-90)/P(0-10)$ as a function of the initial liquid fraction. This ratio is a simple measure to quantify the horizontal and vertical alignment of the fibres which is sensitive to the compressive modulus as we will show in the next section.

The increased flow of liquid (foam drainage) in the initial stage of the fibre-foam dispersion leads to more fibres being aligned towards the direction of drainage. This shows we can control the fibre orientation distributions of the fibre networks simply by varying the liquid fraction of the fibre-foam dispersions. In the next section we show that this is of relevance to the compressive strength of a sample which we can fine-tune.

**Fig. 14:**
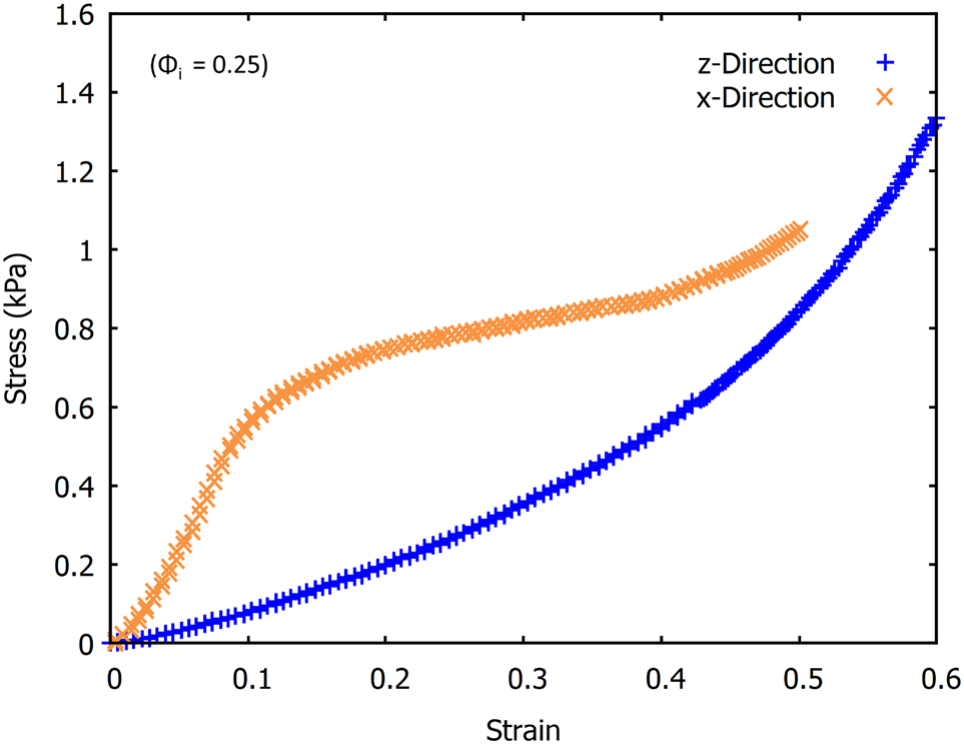
The stress-strain response of the fibrous samples is markedly different in the x and z directions. Compression in the x-direction features a linear stress-strain regime, followed by a plateau. Compression in the z-direction features a steady non-linear increase in stress. The contrasting behaviour can be attributed to the orientations of the fibres and structural layering relative to the direction of compression

## Behaviour under compression

To probe the effect of fibre orientation on compressive strength of our samples, we subjected each to uniaxial compression from all three axial directions. Testing was performed with an Anton Paar MCR301 rheometer, set in a plate-plate configuration. The upper plate was lowered at a rate of 1 mm/min and the resulting stress and strain was measured. The sample size was 33 x 33 x 16mm. In the z-direction the contact area was 33 x 33mm. When a load was applied orthogonal to the direction of drainage the sample was cut beforehand to a height of 16mm, giving a contact area of 16 x 33mm. This was to ensure the maximum compressible height of all samples was the same (i.e. all samples had a height of 16mm, regardless of the direction of compression).

**Fig. 15:**
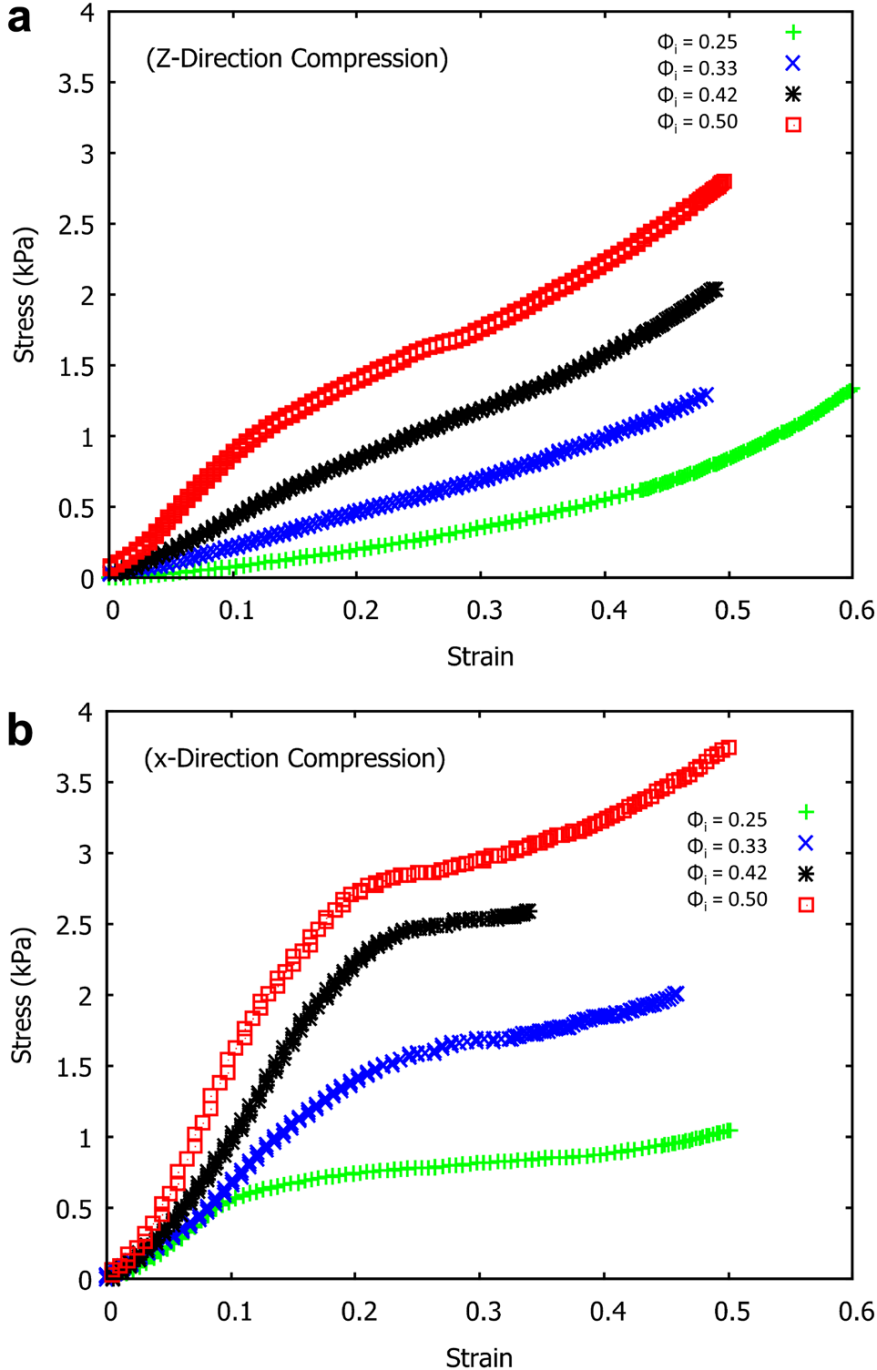
Stress-strain curves of the four fibrous samples (same density but made of different values of $\phi _{i}$) under uniaxial compression. **a** Application of load in the direction of drainage (z-direction). **b** Application of load in the x-direction, orthogonal to drainage, results in higher values of stress

Figure 14 shows that the stress-strain response of our samples depends crucially on the direction of compression, i.e. the samples are highly anisotropic. Compression from the z-direction features a short initial linear regime (more evident in Fig. 15a, followed by a superlinear increase in stress corresponding to densification. When the sample is compressed along the x-direction the stress-strain curves display three distinct regimes. The first is the linear elastic regime which has a higher modulus compared to the z-direction. Between strain of 0.1 and 0.4, very little stress is required to compress the structure, which is followed by sample densification with an increasing number of fibre-fibre contacts being formed.

The stress-strain curves of four fibrous structures of similar density, but produced with different values of $\phi _{i}$ are shown in Fig. 15. For the samples in Fig. 15a the load was applied in the z-direction (direction in which the liquid drained from the samples) and for the samples in Fig. 15b the load was applied in the x-direction. The compressive modulus of elasticity, $E_{c}$, was obtained from the initial linear part of the stress-strain curves, up to a maximum deformation of 0.15.

**Fig. 16:**
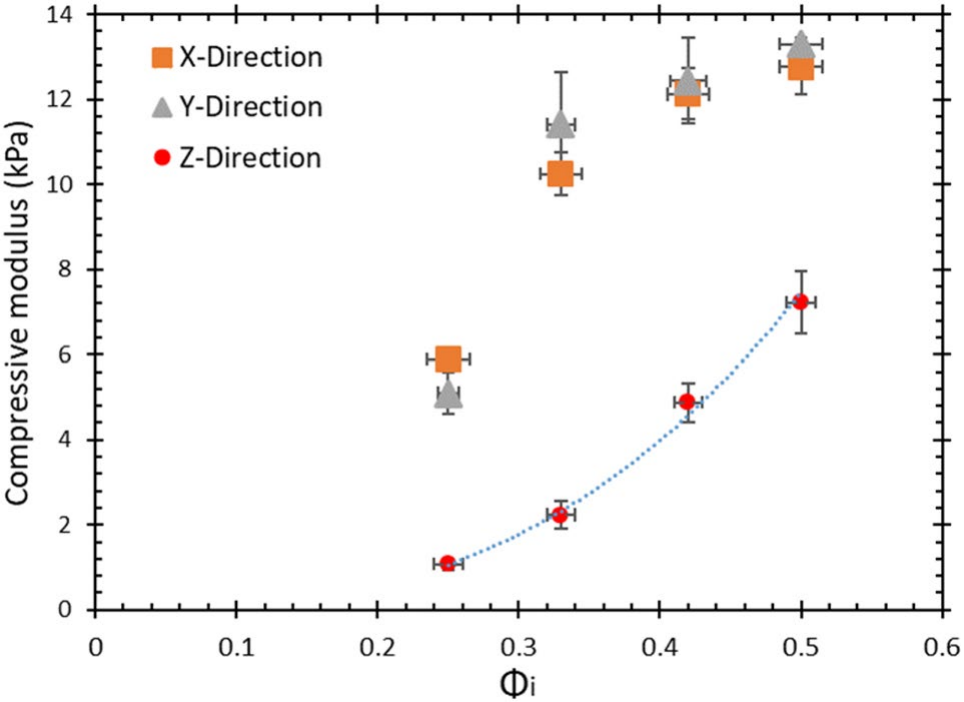
Compressive modulus, $E_{c}$, obtained from the initial linear regime of Fig. 15a and b. $E_{c}$ is seen to increase with $\phi _{i}$ in all three directions

Figure 16 shows an increase of $E_{c}$ with $\phi _{i}$ for all of the data, irrespective of the direction of compression. The x and y directions display very similar results, consistent with results of the fibre orientation analysis and expected from symmetry. The increase of $E_c$ with $\phi _c$ when compressed along the z-direction can be attributed to the larger fraction of fibres orientating into the direction of compression (z-direction) as the initial liquid fraction is increased (see Sect. 3.3). In Sect. 5 we offer an explanation as to why $E_{c}$ increases with $\phi _{i}$ also when compressing in the x and y-directions.

**Fig. 17:**
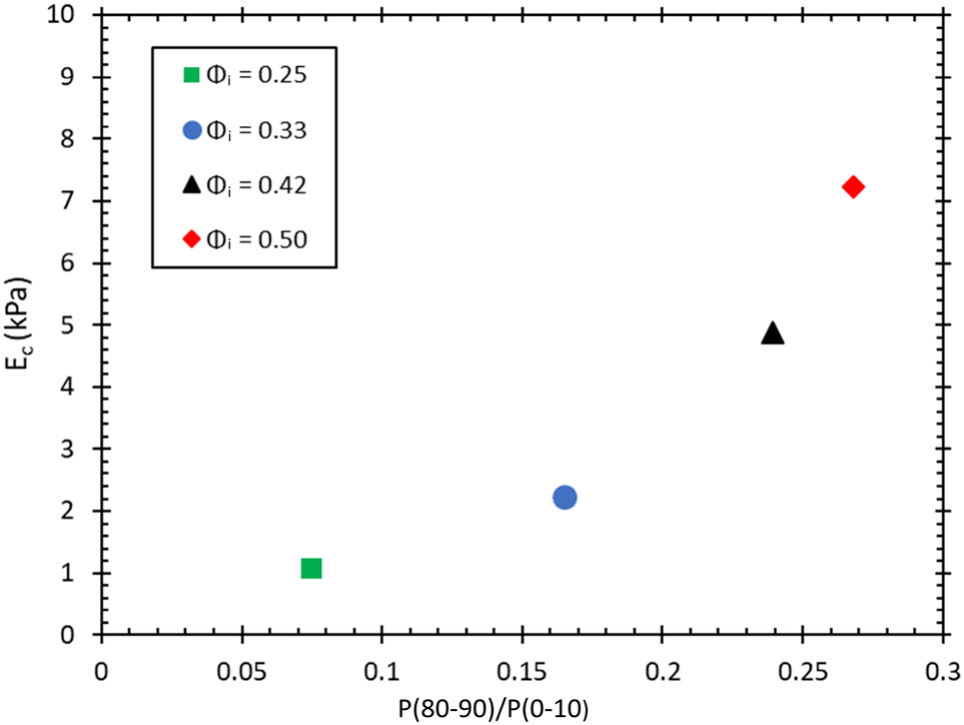
Compressive modulus, $E_{c}$, of the four samples as a function of $P(80-90)/P(0-10)$ (Fig. 13c). The orientation of the compression was in the z-direction (drainage direction). We can see that as the ratio $P(80-90)/P(0-10)$ increases the compressive modulus of the samples is also increased

Figure 17 shows a seven-fold increase of the compressive modulus, as measured along the z-direction. This is solely due to changes in the structure of the fibre network as reflected in the ratio $P(80-90)/P(0-10)$. An increase of this ratio means that fibres are increasingly aligned along the z-direction. This demonstrates the role that the initial liquid fraction plays in the formation of the fibre network structure; increased drainage leads to fibre alignment along the z-direction (direction of gravity). This in turn has a large effect on the compressive modulus while the final density of the structure remains the same.

## Discussion

In the previous sections, we have described the strong anisotropy of both the structure and compression properties of the low-density fibrous samples. In particular, the compressive moduli are much larger in x and y-directions than in the z-direction (direction of drainage). The z-directional modulus increases continuously with initial liquid fraction of the foam precursor, whereas changes in x and y-directional moduli saturate at the higher initial liquid fractions (0.42 and 0.50). Moreover, the monotonic stress-strain behaviour observed for the z-direction deviates qualitatively from the stress plateaus seen at moderate strains for the x and y-directional compression.

In order to explain this anisotropy in the mechanical behaviour, one has to consider structural heterogeneity and anisotropy at different levels. These include the layering of the fibre network (Fig. 12), the cellular porous structure within each layer (Figs. 9 and 11), and the z-directional fibre bundles and segments of single fibres, binding the layers together (Figs. 12 and 13). Below, we consider the possible effects of these three structural features.

For the compression in z-direction, one would expect fibre segments or their bundles, which bridge the layers together, to first bend and buckle, compressing the layers onto one another (see Fig. 12). According to Ketoja et al. [16], when a random fibre network is compressed, the buckling continues throughout the compression cycle, up to the densification regime. This results from an exponential distribution of fibre-segment lengths, valid also for flocculated networks. Fibre segments having the largest free-span length buckle first, distributing their stress onto the remaining fibre segments. These segments then require an increased load to buckle due to their shorter mean free span, resulting in the deformation behaviour for the two lowest liquid fractions, 0.25 and 0.33 as seen in Fig. 15a. Here the relative number of z-directional segments is rather small (see Fig. 13a), and they probably consist mainly of single fibres or small bundles.

However, at the two higher liquid fractions and strains below 0.5, the stress increases more rapidly than the theory by Ketoja et al. would predict. In these cases the segment orientation distribution gets broader (Fig. 13a) due to the larger volume of liquid that had drained during sample preparation. It is possible that this causes the cellular porous structure seen in xy-plane (Fig. 9) to extend also partly in the z-direction. Gibson and Ashby considered the compression of cellular structures and found that the individual columns buckle when the applied force, $F_{c}$, reaches a critical level [17] given by Euler’s formula

$$F_{c}=\mu \frac{\pi ^{2}EI}{a^{2}}$$

Here *E* is the elastic modulus, *I* is the cross-sectional moment of inertia, *a* is the free-span length of the column and $\mu$ is a pre-factor which depends on the boundary conditions at the column ends. Effectively, this is the same formula as for fibre buckling [16], but the distribution of column lengths for the more correlated cellular structure probably differs from the exponential distribution of the fibre segments. Kim *et.al.* have shown fibrin networks behave similar to cellular solids, displaying a buckling regime in their stress-strain response to compressive loading. Evaluating the stress-strain behaviour they obtained the value of critical stress required to initiate the buckling regime in the network [18]. Zhao *et.al.* implement Eulers formula to determine the critical free span length responsible for the transition between the elastic to buckling regime in sintered metal sheets [19].

**Fig. 18:**
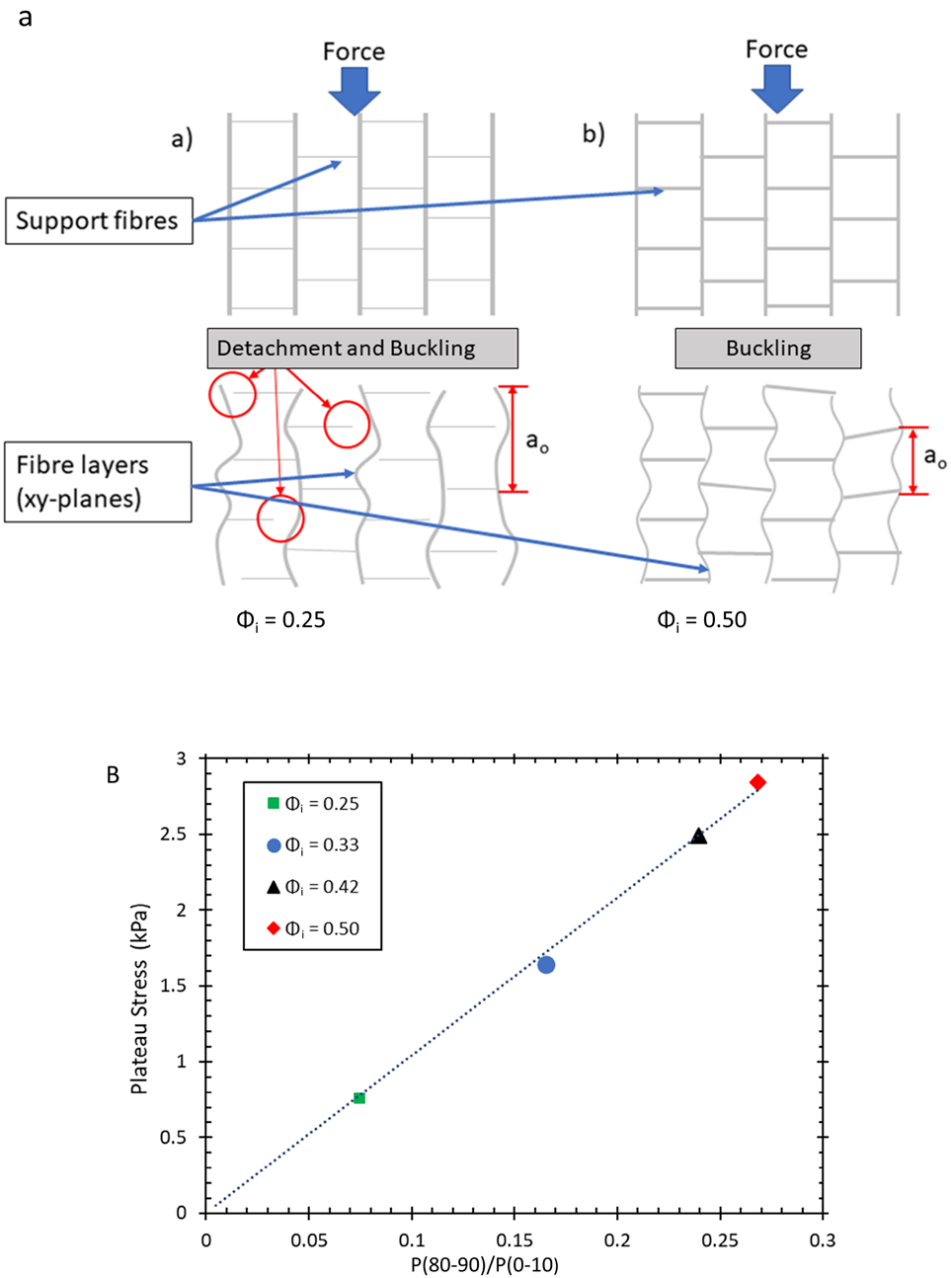
**a** The schematic above gives an explanation for the increase we find in the plateau stress when the samples are compressed in the x and y-directions. The response originates from columnar buckling [20] of the fibre layers in the xy planes. The higher number of support fibres/bundles between the layers increases the critical buckling force and shortens the plateau region as there is less open space between the layers. **b** The Plateau stress varies linearly with the orientation factor $P(80-90)/P(0-10)$, which is a measure of the number of fibres aligned towards the z-direction (see Fig. 13**c**, and which is controlled via the initial liquid fraction $\phi _{i}$ of the precursor fibre-foam dispersion

The much higher stress required for deformation in the x or y-directions (see Figs. 14 and 15b) indicates that the larger structural elements are responsible for the failure; these would be the layers of the fibres in the xy planes rather than single fibre segments [20]. The stress-strain curves display three distinct regimes, the first is a linear elastic regime, followed by a transition to a plateau where layer buckling takes place, then a sharp increase into the densification regime. The range of strains over which the plateau occurs decreases with an increase in $\phi _{i}$, because the gap between the layers gets more narrow as the orientation distribution of the fibres broadens. The z-directional fibre bundles also act as supports that prevent layer buckling, which further enhances the buckling stress with increasing $\phi _{i}$, as shown in Fig. 18. In fact, Fig. 18b shows that the Plateau stress varies linearly with the orientation factor $P(80-90)/P(0-10)$ of Fig. 13c. After the buckling plateau, the onset of densification can be seen in Figs. 14 and 15b (top data set). Interestingly, carbon nanotubes display a similar stress-strain behaviour; when loaded under uniaxial compression buckling takes place in the plateau regime, evident also from SEM images of the rods [20].

## Conclusions

We have analysed both the foam-forming process and the resulting low density fibrous materials: the presence of Kraft fibres inside a foam eventually arrests bubble growth due to gas diffusion. The maximum bubble size was found to be independent of initial liquid fraction $\phi _{i}$. Image analysis of $\mu$CT scan data revealed that the average void size of the fibrous samples was $50\%$ larger than the average bubble size in the precursor foam possibly due to bubble coalescence. Both average void and bubble size are independent of the initial liquid fraction $\phi _{i}$.

Density profiles of the samples showed the layering of fibres in the planes perpendicular to the direction of drainage in the fibre-foam dispersion. Fibre orientation analysis has shown that the majority of fibres lie in these xy-planes, with fewer fibres supporting the layers, i.e. fibres that are more aligned to the direction of gravity. Increasing the initial liquid fraction of the foam $\phi _{i}$ increases the fraction of support fibres; it also has the effect of increasing the compressive modulus of the fibrous material, which could be further increased by the addition of polymers or fibrils [7].

Our results will allow for a greater control of the mechanical properties of these fibrous materials as average void size, fibre orientations and thus the compressive strength of the samples can be tuned via the properties of the fibre-foam dispersions.

## Figures and Tables

**Fig. 19:**
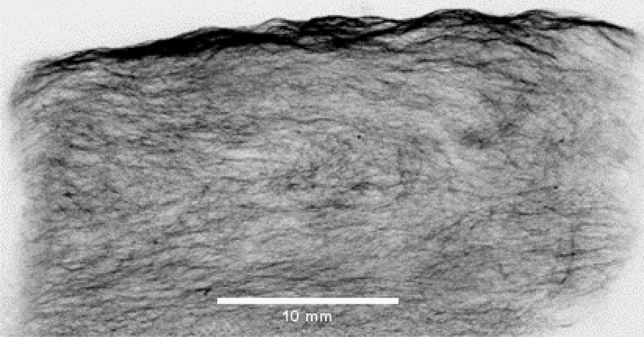
One of 3141 X-ray images used to reconstruct three orthogonal image stacks, as well as three dimensional renderings of the samples. The low beam attenuation of the samples was increased prior to any imaging by coating the individual fibres using an iodine sublimation process

## Appendix A: Details of CT scanning procedure

The fibrous samples were placed in sealed glass containers with a small quantity of iodine crystals before imaging [21]. At room temperature, the iodine crystals sublimate and after approximately four to five days, the iodine vapour that penetrated throughout the porous fibre network leaves an iodine coating on the fibres. This increases the atomic density of the fibres, vastly increasing beam attenuation. The broader spread in the X-rays having passed through the samples, gives rise to higher contrast images, allowing for the detection of individual fibres.

Samples were placed onto a stage located between the target material and a flat panel detector (Varex 1620). The ratio of the distance between the detector and the source, and the source to the sample gave a geometric magnification of 19.7. The resulting effective pixel size, determined by the actual pixel size/ geometric magnification was 10$\,\mu$m. The pixel size of the detector was 200$\,\mu$m set in a 2000 x 2000 pixel matrix. The flat panel detector consists of a layer of scintillator on top of an array of photosensitive diodes. As the X-ray energies are absorbed by the scintillator they are then re-emitted as visible light, which is detected by the photo-diodes and then converted into greyscale images. The sample stage rotates in increments (dependent on the number of X ray images required) through 360 degrees while X-ray images are obtained, an example is shown in Fig. 19.

For our scans the sample stage was rotated in 0.115 degree increments in a stop-start fashion. 3141 X-ray images of each sample were obtained, with an exposure time of 500 milliseconds per image projection and 4 frames per projection, resulting in a total acquisition time of around 90 minutes.

After acquiring the X-ray images, the files are reconstructed into a volume with Nikons CT Pro 3D software using a back-filtered projection algorithm, with a voxel resolution of $10 \, \mu m$ (fiber diameter was approximately $35 \, \mu m$). The files were imported into the visualisation and analysis software, Volume Graphics Studio Max [22].

The software allows a visualisation of the volumes in three dimensions. Voxel grey values are presented in a histogram that displays two distinct peaks, one peak represents the background value and the other is the grey scale value of the material (fibres). Image contrast is adjusted using the opacity curve tool on the histogram, enabling the fibres to become more visible when rendering the volumes. The fibre surface was determined using Volume Graphics surface determination tool, first the background is defined by selecting a part of the image which is clearly the background (above the sample). Next, the material surface is defined by highlighting an area on the fibre. The software then thresholds the volume and displays a 3D rendering for visualisation. The 3D rendering is visually inspected by rotating and zooming through (fly through) the volume to check that the thresholding has not removed any of the connecting fibres, or left behind any artifacts.

Three orthogonal, two-dimensional image stacks of the volumes were exported for analysis. The image stacks represent cross sectional images of the samples, sliced along three orthogonal axis. Before any analysis is carried out, the images were cropped to remove any edge effects that may have occurred when cutting the sample to size . We also exclude the very top and bottom of the sample. As a result, we probed a cube (10mm in edge length) from each sample centre (see Fig. 2).

## Appendix B: OrientationJ analysis

We use the OrientationJ plugin to compute the orientation of the fibres/cell walls in the 2D image slices. The user specifies a window size, which is of the order of the typical fibre width, the coherency threshold and the so-called energy. Specifying the coherency level allows us to filter out the noise in the image, while specifying the minimum energy allows us to filter out pixels that are not close to an edge. Details of the algorithm and the correct choice of parameters can be found in [15].

The interrogation window size was set to 3 pixels, which corresponds to the average fibre width.

The energy level is used to identify image gradients. Low energy levels imply that there is little or no change in the gradient between the adjacent pixels, represented by the directional derivatives having constant values (such as the pixels on the fibre surface and not at the edge). To give a true representation of the distribution of fibre orientations, our analysis only included the orientation of pixels at edge of the fibres (higher energy levels). The lower energy pixels were excluded from the distributions by setting the minimum energy level at 3%, the same level as used by [15] their analysis of the orientation distribution of collagen fibres.

The analysed images also contained fibres orientating out of the image plane (appearing as small clusters of white pixels against a black backgroud). Fibres projecting out of the plane have a low coherency level. We have excluded these fibres, and any noise present in the images from the analysis, by setting the minimum coherency level at 10%.

We note that the observed trend, namely that the vertical alignment of fibres increases with initial liquid fraction, is robust with respect to small changes in the OrientationJ parameters mentioned above.
